# Mechanistic Evaluation of a Novel Small Molecule Targeting Mitochondria in Pancreatic Cancer Cells

**DOI:** 10.1371/journal.pone.0054346

**Published:** 2013-01-21

**Authors:** Yumna H. Shabaik, Melissa Millard, Nouri Neamati

**Affiliations:** Department of Pharmacology and Pharmaceutical Sciences, School of Pharmacy, University of Southern California, Los Angeles, California, United States of America; Wayne State University School of Medicine, United States of America

## Abstract

**Background:**

Pancreatic cancer is one of the deadliest cancers with a 5-year survival rate of 6%. Therapeutic options are very limited and there is an unmet medical need for safe and efficacious treatments. Cancer cell metabolism and mitochondria provide unexplored targets for this disease. We recently identified a novel class of triphenylphosphonium salts, TP compounds, with broad- spectrum anticancer properties. We examined the ability of our prototypical compound TP421– chosen for its fluorescent properties – to inhibit the growth of pancreatic cancer cells and further investigated the molecular mechanisms by which it exerts its anticancer effects.

**Methodology/Principal Findings:**

TP421 exhibited sub-micromolar IC_50_ values in all the pancreatic cancer cell lines tested using MTT and colony formation assays. TP421 localized predominantly to mitochondria and induced G_0_/G_1_ arrest, ROS accumulation, and activation of several stress-regulated kinases. Caspase and PARP-1 cleavage were observed indicating an apoptotic response while LC3B-II and p62 were accumulated indicating inhibition of autophagy. Furthermore, TP421 induced de-phosphorylation of key signaling molecules involved in FAK mediated adhesion that correlated with inhibition of cell migration.

**Conclusions/Significance:**

TP421 is a representative compound of a new promising class of mitochondrial-targeted agents useful for pancreatic cancer treatment. Because of their unique mechanism of action and efficacy further development is warranted.

## Introduction

Pancreatic cancer is the fourth leading cause of cancer related deaths in the United States with an overall 5-year survival rate of 6% [1]. Since 2005, the standard chemotherapeutic treatment is the administration of gemcitabine, a nucleoside analog, combined with erlotinib, a kinase inhibitor [2], [3]. Gemcitabine targets ribonucleotide reductase causing depletion of dNTPs and further gets incorporated into DNA causing a stall in synthesis [4]. On the other hand erlotinib, originally thought to target epidermal growth factor receptor (EGFR), has recently been documented to be a multi-kinase inhibitor [5]. The pathway for gemcitabine activity is sufficiently complicated, including uptake transporters and intracellular phosphorylation leading to cytotoxicity, which contributes to the low rate low rate of response in patients and the increasing development of chemoresistance [6]. It has recently been proposed that PDAC stratification into multiple subtypes based on molecular differences can determine response to chemotherapy [7]. Two of the three defined subtypes are represented among the commonly used pancreatic cancer cell lines, including MIA PaCa-2, PANC-1 and HPAC which we utilized in our study.

Among the earliest molecular changes underlying pancreatic cancer is a constitutively activating K-ras mutation that occurs in nearly 100% of cases [8], [9]. During transformation, K-ras signaling drives excessive cell proliferation and promotes survival. It has been proposed that mitochondrial energy production is essential in supporting Ras-transformed cells that become heavily reliant on autophagy, a state referred to as “autophagy addiction”, to maintain a healthy pool of mitochondria and sufficient TCA cycle intermediates to support oxidative phosphorylation (OXPHOS) [10], [11]. Notably, in pancreatic cancer cell lines and patient samples, the basal level of autophagy is elevated as compared to normal cells or cells from other tumor cell lines and is correlated with poorer clinical outcomes [10], [12]. This phenotype, characteristic of Ras-transformed cells, makes them uniquely susceptible to disruption of mitochondrial respiration and autophagy. In fact, pharmacological inhibition as well as silencing of key autophagy genes has successfully resulted in reduction of mitochondrial oxygen consumption and intracellular ATP levels leading to profound inhibition of pancreatic cancer growth both in vitro and in vivo [10]. Therefore, inhibition of autophagy and mitochondrial targeting could provide a new approach for treating PDACs that are usually highly refractory to available chemotherapies. Indeed, there has been a recent surge in interest for targeting cancer cell mitochondria following the recognition of their altered bioenergetic status as a contributor to cancer pathogenesis [13]. Consequently, targeting mitochondria has emerged as a new ideal for anticancer therapy aided in part by the knowledge of achieving precise delivery of drugs to the organelle. The use of mitochondrial targeted agents for anticancer therapies presents an added benefit of directly acting upon the main regulator of programmed cell death within the cell and entirely bypassing the upstream signaling cascades that are often undermined [14]. It has been well documented that mitochondria of malignant cells exhibit a higher transmembrane potential as compared to non-malignant cells with differences in enzyme activities, electron carriers and membrane lipid structure as potential underlying causes [15]. Exploiting this unique attribute has led to the design of novel lipophilic cations that can preferentially accumulate in tumor cell mitochondria over normal tissue driven by the increased transmembrane potential [15]. Conjugation of triphenylphosphonium (TPP) cations to a variety of chemical probes and drugs is widely used to achieve specific targeting to the mitochondria [16], [17], [18], [19], [20]. A TPP-tagged vitamin E analog (MitoVES) has been extensively studied for its apoptotic and anti-angiogenic properties, which are far enhanced over that of the parental un-tagged compound, supporting mitochondrial targeting as a viable approach for improving efficacy of moderately active drug candidates [20], [21], [22]. Alternatively, drug delivery carriers such as dendrimers and lipososmes have been modified with TPP cations and utilized to deliver cargo to mitochondria [17], [23], [24], [25]. When these carriers were loaded with model chemotherapeutic drugs including paclitaxel or doxorubicin, efficacy was enhanced over non-targeted carriers indicating that delivery to the mitochondria can improve apoptosis and cytotoxicity.

We have previously reported the discovery of a novel class of TPP salts that have broad-spectrum anticancer activity [26]. These compounds can accumulate in mitochondria by virtue of their positively charged TPP moieties and the negative potential across the mitochondrial membranes [27]. Subsequently, we have demonstrated that these compounds are capable of abrogating mitochondrial respiration and increasing mitochondrial superoxide levels. Most importantly, we have shown that this class of compounds can effectively inhibit tumor growth in a nude mouse breast cancer xenograft model with no apparent toxicity [26]. Because of their unique mechanism of action targeting mitochondria and the exquisite dependence of Ras-driven tumors on mitochondrial function, we sought to develop this class of compounds as novel agents against pancreatic cancer. Furthermore, given the importance of autophagy to maintain a healthy pool of mitochondria for supporting survival and proliferation of Ras transformed cells, we hypothesized that TP421 would induce cell death in pancreatic cancer cells in a manner involving autophagy regulation of mitochondria.

## Materials and Methods

### Cell Lines and Culture Reagents

MIA PaCa-2, PANC-1, BxPC-3 and HPAC pancreatic cancer cell lines were purchased from the American Type Culture Collection (ATCC; Manassas, VA). MEF atg3+/+ and atg3−/− murine cell lines were a gift from Masaaki Komatsu (Juntendo University School of Medicine, Bunkyo-ku, Tokyo) [28]. HFF-1 normal fibroblast cell line was provided by Dr. Carla Grandori (Fred Hutchinson Cancer Research Center, Seattle, WA). All cell lines used for experimentation were maintained in culture under 35 passages and tested regularly for mycoplasma contamination using PlasmoTest™ (InvivoGen, San Diego, CA). MIA PaCa-2 and PANC-1 cells were maintained in DMEM supplemented with 10% fetal bovine serum (FBS; Gemini-Bioproducts, West Sacramento, CA). BxPC-3, MEF atg3+/+ and atg3−/− cells were maintained in RPMI supplemented with 10% FBS. HPAC cells were maintained in 1∶1 mixture of DMEM and Ham’s F12 medium supplemented with 5% FBS. Cells were grown at 37°C in a humidified atmosphere of 5% CO2. For all experiments, cells in exponential growth phase were rinsed with DPBS without calcium and magnesium, briefly trypsinized in a small volume of 0.25% trypsin-EDTA solution (Sigma-Aldrich, St. Louis, MO), re-suspended in complete culture media, manually counted and seeded on to sterile plates and allowed to adhere overnight before treating.

### Compounds

All compounds were stored as concentrated DMSO stock frozen at -20°C. Dilutions were prepared in DPBS, without calcium and magnesium, and re-used for a maximum of 3 freeze-thaw cycles. Compounds were purchased from LKT laboratories Inc. (St. Paul, MN), Sigma-Aldrich Corp. (Saint Louis, MO) and Asinex Ltd. (Moscow, Russia).

### Cytotoxicity Assay

Cytotoxicity was measured by the 3-(4,5-dimethylthiazol-2-yl)-2,5-diphenyltetrazolium bromide (MTT) colorimetric assay as previously described [26].

### Colony Formation Assay

Cells were seeded in 6-well plates (500–1000 cells per well depending on the cell line) and allowed overnight to adhere. The next day, drugs were added to the wells for 24 h following which media was replaced with drug free media. Cells were further incubated for 7–14 days to allow colonies to form. Colonies were stained with a crystal violet solution (0.05% crystal violet, 0.74% formaldehyde and 36% methanol), rinsed and then imaged.

### Trypan Blue Exclusion Assay

Treated cells were collected via brief trypsinization and stained using trypan blue 0.4% solution (Lonza Group Ltd) according to manufacturer’s recommendation. Cells were counted using a hemacytometer.

### Alamar Blue Cell Viability Assay

Cells were handled identical to MTT assay described above. At the end of treatment, alamar blue (AbD Serotec^©^) was added to wells according to manufacturer’s recommendation. Fluorescence was detected at 560/590 ex/em wavelengths.

### Cell Cycle Determination

Drug treated cells were collected via brief trypsinization, and fixed in 70% ethanol at −20°C for at least 4 h. For determining DNA content, samples were spun down and re-suspended in DPBS containing propidium iodide (50 µg/mL final concentration) and RNase A (100 µg/mL final concentration) and analyzed by flow cytometry.

### Cell Lysates and Western Blotting Analysis

Treated cellswere rinsed with DPBS and lysed on ice by addition of a small volume of SDS-containing cell lysis buffer for 15 min. Lysates were collected in eppendorf tubes, sonicated on ice to shear DNA, and quantified using the RC DC protein assay (Bio-Rad Laboratories, Hercules, CA). Thirty micrograms of cell lysate was loaded in each lane and subjected to SDS-PAGE and subsequently transferred to Immun-Blot polyvinylidene fluoride membranes (PVDF; Bio-Rad Laboratories, Hercules, CA). Briefly, membranes were blocked in 5% milk in TBST, followed by incubation in primary antibody diluted in appropriate blocking buffer at 4°C overnight.After, membranes were washed, appropriate secondary antibody was added for 1 h at room temperature and finally washed off before imaging. All antibodies were purchased from either Cell Signaling Technology Inc. (Danvers, MA) or Santa Cruz Biotechnology Inc. (Santa Cruz, CA). Protein detection was carried out using Super Signal West Dura chemiluminescent substrate (ThermoFisher Scientific, Waltham, MA) and imaged on ChemiDoc™ XRS+ system (Bio-Rad Laboratories, Hercules, CA). For all western blot data, images are representative blots chosen from at least two independent experiments.

### Measurement of Hydrogen Peroxide Levels

The generation of hydrogen peroxide was measured using an Amplex Red enzyme assay. Cells seeded in 96-well clear bottom black plates were treated for 4 h with the desired concentration of drug in phenol red-free DMEM medium supplemented with 10% FBS. At the end of treatment, cells were washed and lysed in DPBS buffer containing 50 µM Amplex Red (Molecular Probes) and 0.1 units/mL horseradish peroxidase (HRP; Sigma, St. Louis, MO) and incubated for 30 min at 37°C, protected from light. Following incubation, fluorescence intensity of each well was detected at 540/590 ex/em wavelengths.

### Measurement of Superoxide Anion Levels

Drug treated MIA PaCa-2 and BxPC-3 cells were briefly stained with MitoSOX Red Mitochondrial Superoxide Indicator (Invitrogen, Carlsbad, CA) following manufacturer’s recommendations.Fluorescence intensity was measured using flow cytometry as previously described [26].

### Fluorescent Microscopy of TP421 Subcellular Localization

MIA PaCa-2 and PANC-1 cell lines were seeded in double chambered cover glass (Nalge Nunc International, Rochester, NY) at a density of 50,000 cells/chamber and allowed overnight to adhere. The following day, the cells were treated with 2 µM TP421 for time periods up to 72 h. Prior to imaging, cells were stained for 15 minutes at 37 °C using either 200 nM Mitotracker Red CMXRos or 50 nM Lysotracker Red DND 99 live cell organelle stains (Life Technologies, Grand Island, NY)following manufacturer recommendations. Live cells were visualized using a Nikon DAIPHOT 300 inverted microscope (Nikon Instruments, Melville, NY) equipped with DAPI and Cy3 filter blocks, 10x eye piece and 100x/1.3 Nikon oil immersion lens and super high pressure mercury lamp. To prevent photobleaching, sample exposure to light was minimized during image acquisition by engaging neutral density (ND2 and ND4) filters to limit the intensity of light reaching the specimen. Images were captured using a Photometrics CoolSNAP 9 CCD camera (Roper Scientific, Ottobrun, Germany) and processed using Q-capture Pro v 5.1.1.14 imaging software (Q imaging corporation, Surrey, BC, Canada).

### Immunofluorescent Staining of LC3B

MIA PaCa-2 cells were seeded on glass coverslips at a density of 50,000 cells and allowed overnight to adhere. The following day, cells were treated with 2 µM TP421 in the presence or absence of 5 µM rapamycin or 10 µM chloroquine for 18 h. At the end of treatment, media was removed and cells were washed with 500 µL DPBS prior to fixation with 3.7% formaldehyde for 15 m at RT. Fixed cells were washed with 500 µL DPBS prior and subsequent to permeablization with ice-cold acetone for 5 minutes at -20C. Coverslips were blocked for 30 min with 1% bovine serum albumin (BSA) in DPBS prior to incubation overnight at 4°C with LC3B antibody in 1% BSA. Antibody was removed and coverslips were washed in DPBS with gentle agitation. Cy5 and Cy3-conjugated antibodies (GE Healthcare Lifesciences, Pittsburgh, PA) diluted in 1% BSA were incubated with coverslips for 2 h. Coverslips were again washed in DPBS with gentle agitation, air-dried and mounted on pre-cleaned glass slidesusing Prolong Gold anti-fade mounting media (Life Technologies, Grand Island, NY). Images were obtained using a Lieca SP2 scanning confocal microscope (Leica Microsystems, Heidelberg, Germany) equipped with 488 nm argon and 633 nM krypton lasers (laser power was kept to a minimum <50% of total) and Leica Confocal software v 2.61 (Leica Microsystems, Heidelberg, Germany).

### In vitro Migration Assay

Cell migration was assayed using 24-well plate cell culture inserts fitted with transparent PET membranes having 8 µm sized pores (BD Biosciences, San Jose, CA). 7.5×10^4^ serum starved MIA PaCa-2 cells were plated in the top chamber in serum free media and allowed to lightly adhere overnight. On the next day, migration was stimulated with 10% FBS medium added to the lower chamber. Negative control wells received 1% serum medium instead. TP421 treated samples, received 10% FBS medium in the lower chamber and compound in serum free medium in the top chamber. After 24 h, cells adherent to the top side of the membrane were lightly scrapped off using a Q-tip. The cells on the bottom side of the membrane were stained using giemsa nuclear stain for 30 minutes at room temperature and washed with deionized water. Images of the stained membranes were captured from representative fields using Nikon inverted microscope using a 10X objective.

### Wound Healing Assay

Tissue culture treated 96-well plates were coated with collagen I dissolved in 0.2 N acetic acid (sterile filtered) to a final concentration of 45 µg/mL, overnight at 4°C overnight. The following day excess collagen was removed, the wells were washed twice with DPBS and blocked with 2 mg/ml BSA in DPBS for 1 h at room temperature. After blocking, the wells were again washed DPBS. PANC-1 cells were then seeded at a density of 35,000 cells/well in full serum media and allowed to adhere overnight. Media was changed to serum-free media and cells were further incubated overnight. The following day, scratches were made using the edge of a 200 µL tip and wells were washed with DPBS. Treated and un-treated control wells received 10% FBS containing media and drug was added. Negative control wells received serum-free media. Wounds were allowed 24 h to close at the end of which wells were rinsed with DPBS, cells were fixed in 100% methanol for 10 min and stained with giemsa nuclear stain for 30 min. Finally, wells were washed with deionized water and wallowed to dry. Wounds were imaged with a using a 4X objective.

### Statistical Analysis

Where indicated, p-values were calculated using the two-tailed unpaired student’s t-test with unequal variances. P-values less than 0.05 were considered to be statistically significant.

## Results

### TP421 Shows Rapid and Potent Cytotoxicity in a Panel of Pancreatic Cancer Cell Lines

**Figure 1:**
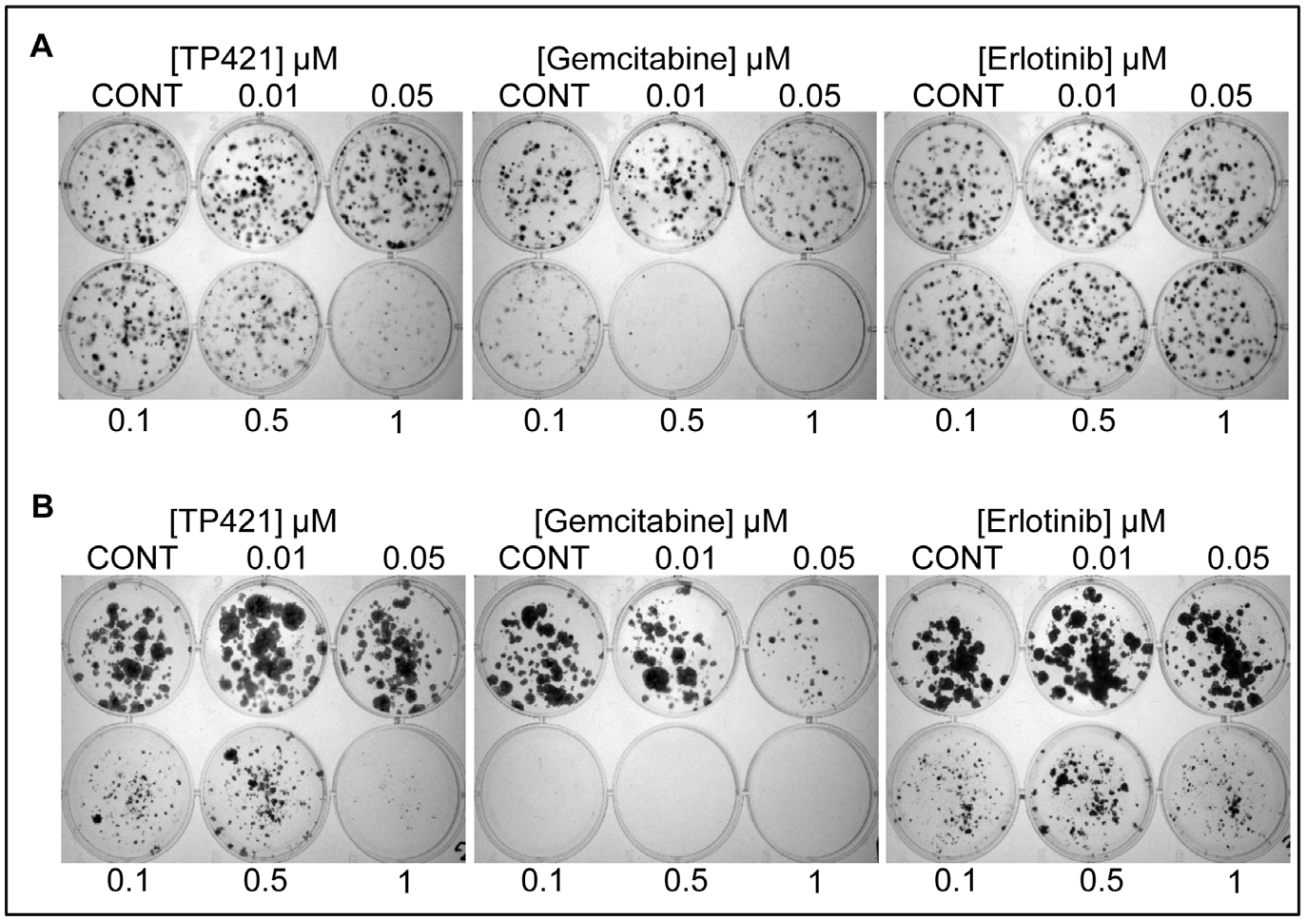
TP421 significantly inhibits colony formation of pancreatic cancer cells. Effect of 24 h drug exposure on colony forming ability of (A) MIA PaCa-2 and (B) BxPC-3. Images are representative of three independent experiments.

**Figure 2:**
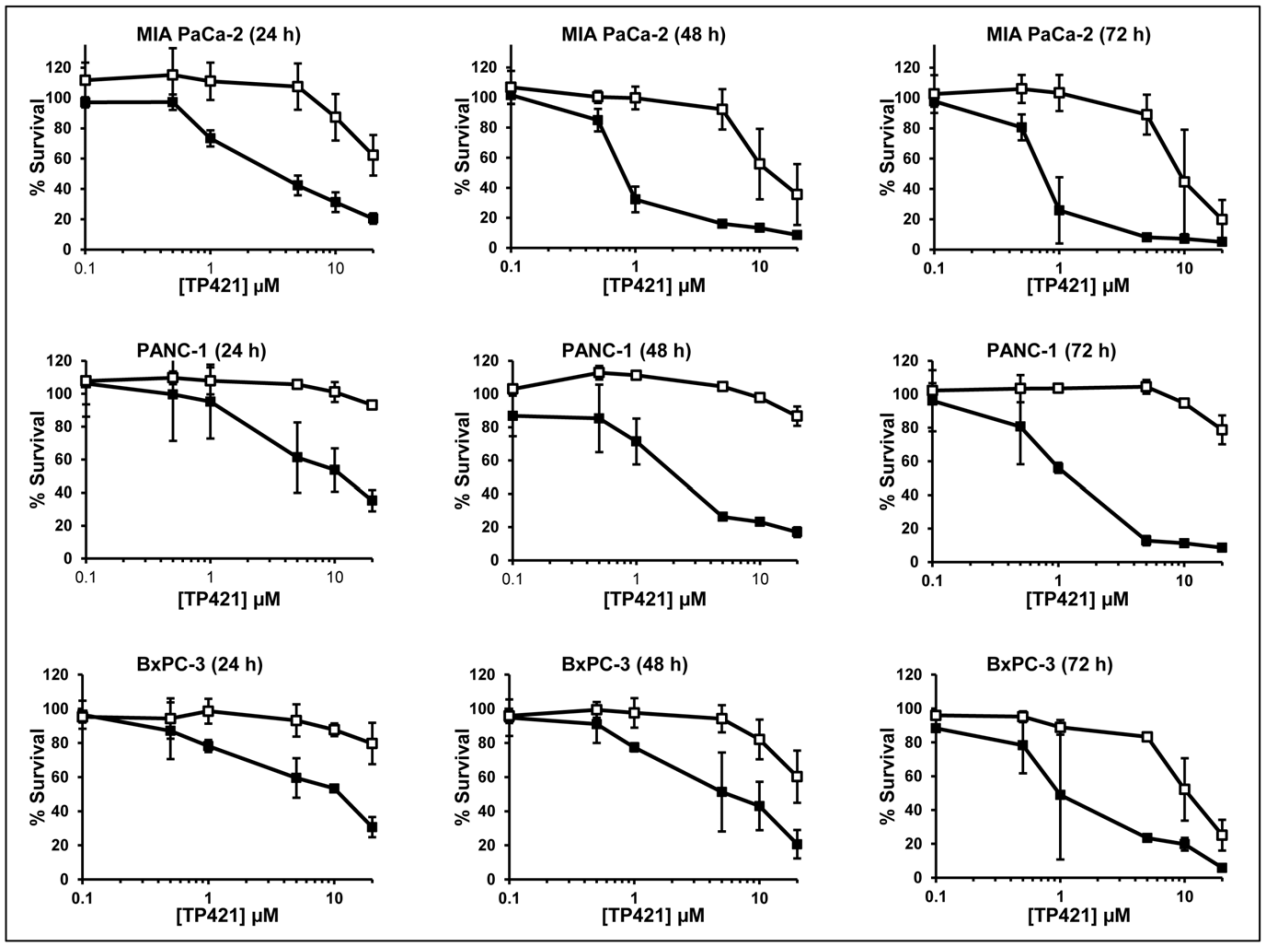
Effect of shorter exposure time on the cytotoxicity of TP421 in pancreatic cancer cells. Dose response survival curves for cell lines treated with TP421 comparing continuous exposure (closed squares) to 0.25 h drug exposure followed by a media wash out (open squares) and further incubation. Total incubation time is indicated in parentheses. The data are mean ± SD from three independent experiments. TP421 concentrations are plotted on a Log base 10 scale.

In our initial report on the anticancer activity of this class of compounds we described their potent cytotoxicity against cancer cell lines of varying lineages [26]. Here in, we tested the ability of one of the lead compounds identified form our initial screen, TP421, to induce cytotoxicity and inhibit cell proliferation in a panel of pancreatic cancer cell lines. Using MTT assay, we evaluated the effect of 72 h continuous exposure to escalating doses of TP421, TP187, and TP197 on the growth of MIA PaCa-2, BxPC-3, PANC-1 and HPAC cell lines. The results (**Table 1**) revealed potent cytotoxicity, with TP421 IC_50_ values in the sub-micromolar range in all of the tested cell lines irrespective of their differences in differentiation state or genetic mutational background. Structures of the compounds tested are shown in the supporting figure (**Figure S1**). The TPP moiety in this compound is essential for activity as 7-diethylamino-4-methylcoumarin, a structurally identical analog of TP421 lacking the TPP moiety, did not produce cytotoxicity in the cell lines tested. We further examined the effect of 24 h exposure to TP421 on the colony forming capability of MIA PaCa-2 cells as compared to gemcitabine and erlotinib (**Figure 1**). The results confirmed the ability of TP421 to inhibit cell proliferation *in vitro* to levels comparable with gemcitabine and far more potently than erlotinib. To further characterize the cytotoxic activities of TP421, we incubated cells in the presence or absence of a range of concentrations of TP421 for 0.25, 0.5, 1 or 5 h, followed by incubation in drug-free media for 24, 48 and 72 h. In parallel, we treated cells with a continuous exposure to TP421 for 24, 48 and 72 h. At the end of incubations we assessed cell viability via MTT. As expected, the IC_50_ of TP421 had an inverse correlation with the treatment time as well as the total duration of incubation time, as shown in **Table 2**. Unexpectedly however, we observed that with very short exposure times, as short as 15 min, TP421 was capable of achieving 50% cytotoxicity albeit at higher concentrations (20 µM) indicating a very rapid initial drug effect. Interestingly, we found that for PANC-1 cells treated in this manner, shorter drug exposure times greatly reduced the cytotoxicity of TP421. The survival curves comparing continuous and 0.25 h exposure times in the three cell lines are shown in **Figure 2**.

**Table 1:** IC_50_ values (µM) for TP421 and close structural analogs in pancreatic cancer cell lines.

Compound	MIA PaCa-2	BxPC-3	PANC-1	HPAC	HFF-1
**TP187**	0.6±0.4	N/A	0.8±0.1	0.9±0.4	N/A
**TP197**	0.2±0.05	N/A	0.6±0.01	1.2±0.7	N/A
**TP421**	0.5±0.3	0.8±0.07	1.1±0.4	0.4±0.02	3.4±0.1
**7-Diethylamino-4-methylcoumarin**	>20	>10	>10	N/A	N/A
**Gemcitabine**	0.07±0.05	0.04±0.02	>20	N/A	N/A
**Erlotinib**	23.75±7.4	70±31.4	165±30.5	N/A	N/A

**Table 2:** IC_50_ values (µM) for TP421 in pancreatic cancer cell lines by duration of drug exposure and length of incubation before addition of MTT reagent.

		Incubation time (h)		
Cell line	TP421 exposure (h)	24	48	72
		**IC50 (µM)**		
**MIA PaCa-2**	Continuous	2.6	0.8	0.9
	5	15	8	6
	1	20	16	10
	0.5	>20	16	11.5
	0.25	>20	20	14
**PANC-1**	Continuous	4	1.7	1.2
	5	>20	>20	>20
	1	>20	>20	>20
	0.5	>20	>20	>20
	0.25	>20	>20	>20
**BxPC-3**	Continuous	5	2.9	2.1
	5	>20	18	5
	1	>20	18	14
	0.5	>20	20	14
	0.25	>20	20	14

### TP421 Cytotoxicity is Selective Towards Cancer Cells

**Figure 3:**
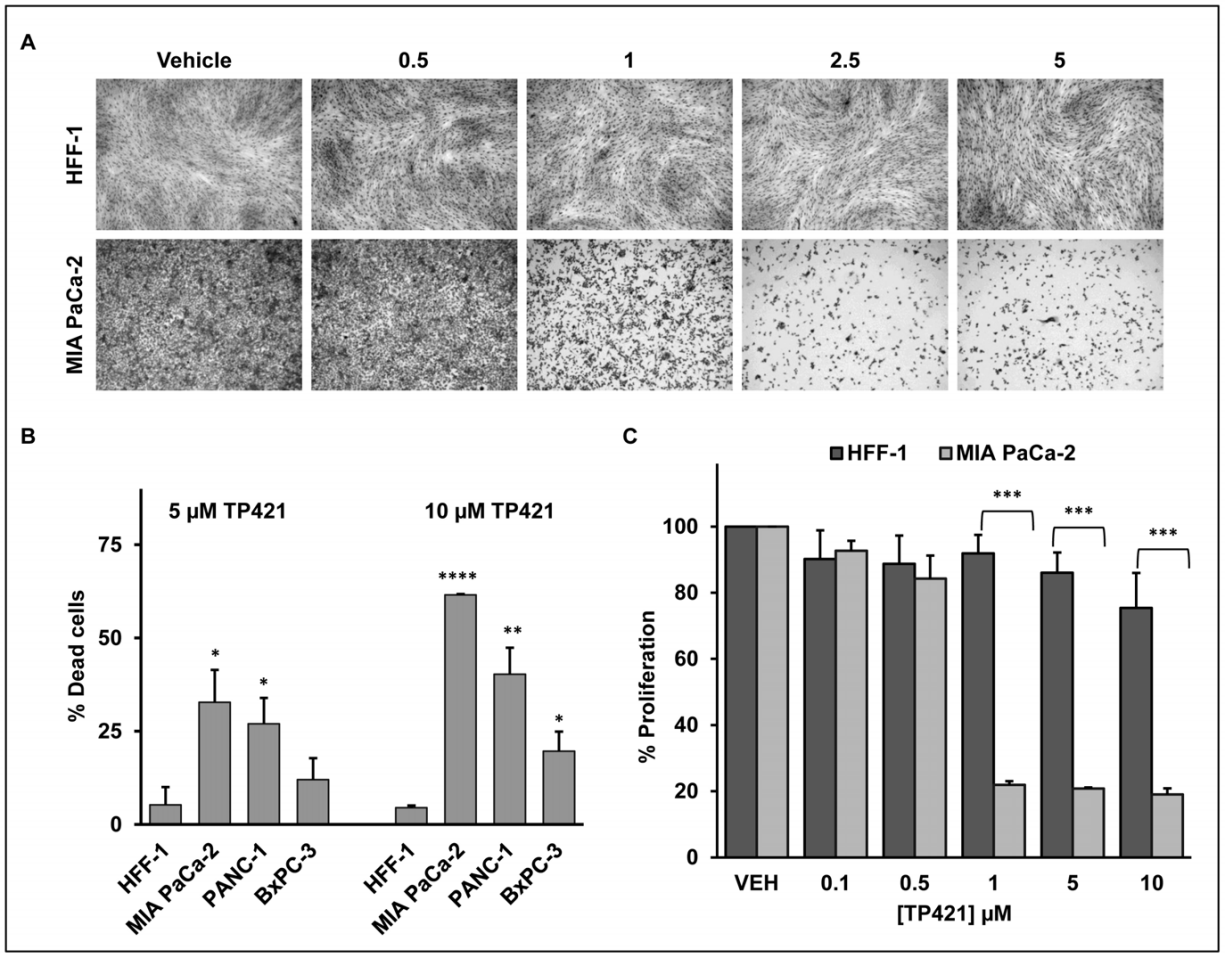
TP421 cytotoxicity is selective for cancer cells. (A) Growth of normal HFF-1 cells is unaffected while the pancreatic cancer MIA PaCa-2 cells show extensive death following 72 h exposure to escalating doses of TP421. (B) TP421 induces greater cell death in three pancreatic cancer cell lines as compared to HFF-1 cells as measured by trypan blue exclusion. (C) Proliferation of MIA PaCa-2 but not HFF-1 is greatly inhibited by TP421 in the alamar blue assay. Three independent experiments were conducted, representative images are shown in (A), mean ± SD are plotted in (B) and (C). *, **, *** and **** indicate p-value <0.05, p<0.01, p<0.001 and p<0.00005 respectively.

Following the assumption that TP421 would selectively accumulate in cancer cells driven by the higher transmembrane potential, we compared the effect of TP421 on the growth characteristics of the normal fibroblast cell line HFF-1 against three of the pancreatic cancer cell lines. Treating MIA PaCa-2 and HFF-1 cells with a range of doses of TP421 for 72 h revealed distinctly higher sensitivity of the pancreatic cancer cell line towards the drug (**Figure 3A**). This was further confirmed by measuring the percentage of dead cells accumulated by TP421 treatment which was significantly higher in the pancreatic cancer cells as compared to HFF-1 (**Figure 3B**). Further, utilizing the alamar blue indicator dye, we observed a 4-fold difference in the inhibition of MIA PaCa-2 cell proliferation as compared to HFF-1 cells following 72 h incubation period with TP421 at the indicated doses. (**Figure 3C**).

### TP421 Arrests Pancreatic Cancer Cell Lines in G_0_/G_1_ Phase of the Cell Cycle in a Time and Dose-dependent Manner

**Figure 4:**
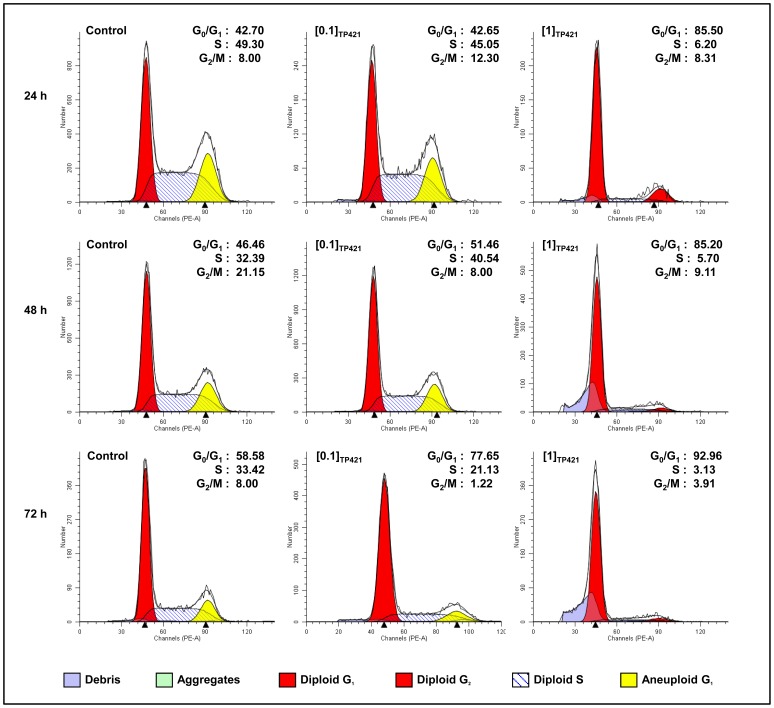
TP421 arrests pancreatic cancer cells in G_0_/G_1_ phase of the cell cycle. The effect of TP421 treatment on the cell cycle distribution of MIA PACa-2 cells was examined in a dose and time-dependent manner. Cells were untreated or treated with 0.1 and 1 µM TP421 for 24, 48 and 72 h. Histograms depicted are representative of three independent experiments.

In order to elucidate the mechanism by which TP421 inhibits cell proliferation, we evaluated its effect on cell cycle kinetics. MIA PaCa-2 (**Figure 4**), PANC-1, BxPC-3, and HPAC (**Table 3**) cells were treated with two concentrations of TP421 for increasing durations of time and DNA content was analyzed using flow cytometry. Extensive G_0_/G_1_ arrest was observed in all the cell lines despite minor differences in their sensitivities to TP421. Interestingly, the cell cycle distribution for MIA PaCa-2 cells treated with the two other analogs, TP187 and TP197, also resembled TP421 with significant and early arrest in G_0_/G_1_ (**Table 3**). This result differs from the G_2_/M and S-phase arrest induced by these compounds in non-pancreatic cancer cell lines [26], indicating a potential tumor-type specific effect.

**Table 3:** Percent distribution of DNA content per cell cycle phase of several pancreatic cancer cell lines in response to treatment with TP421 and close analogs.

		24 h			48 h			72 h			96 h		
Cell line	Sample	G_0_/G_1_	S	G2/M	G_0_/G_1_	S	G2/M	G_0_/G_1_	S	G2/M	G_0_/G_1_	S	G2/M
**MIA PaCa-2**	Control	66.9	29.0	4.1	N/A	N/A	N/A	N/A	N/A	N/A	N/A	N/A	N/A
	2 µM TP421	92.0	8.0	0.0	N/A	N/A	N/A	N/A	N/A	N/A	N/A	N/A	N/A
	2 µM TP187	83.9	16.1	0.0	N/A	N/A	N/A	N/A	N/A	N/A	N/A	N/A	N/A
	2 µM TP197	87.8	9.3	2.9	N/A	N/A	N/A	N/A	N/A	N/A	N/A	N/A	N/A
**PANC-1**	Control	N/A	N/A	N/A	72.2	20.0	8.0	68.5	23.5	8.0	65.8	26.2	8.0
	1 µM TP421	N/A	N/A	N/A	69.4	22.6	8.0	77.8	14.2	8.0	74.5	17.5	8.0
	2.5 µM TP421	N/A	N/A	N/A	68.9	23.1	8.0	74.5	17.5	8.0	73.5	18.5	8.0
**BxPC-3**	Control	N/A	N/A	N/A	54.4	37.6	8.0	69.8	22.2	8.0	N/A	N/A	N/A
	1 µM TP421	N/A	N/A	N/A	79.2	17.8	3.0	82.0	14.6	3.4	N/A	N/A	N/A
	5 µM TP421	N/A	N/A	N/A	83.6	14.8	1.6	80.3	16.9	2.8	N/A	N/A	N/A
**HPAC**	Control	58.4	33.6	8.0	N/A	N/A	N/A	N/A	N/A	N/A	N/A	N/A	N/A
	5 µM TP421	72.6	22.8	4.6	N/A	N/A	N/A	N/A	N/A	N/A	N/A	N/A	N/A
	10 µM TP421	74.5	17.5	8.0	N/A	N/A	N/A	N/A	N/A	N/A	N/A	N/A	N/A

### TP421 Localizes to Mitochondrial Structures with Sustained Retention Over Time

**Figure 5:**
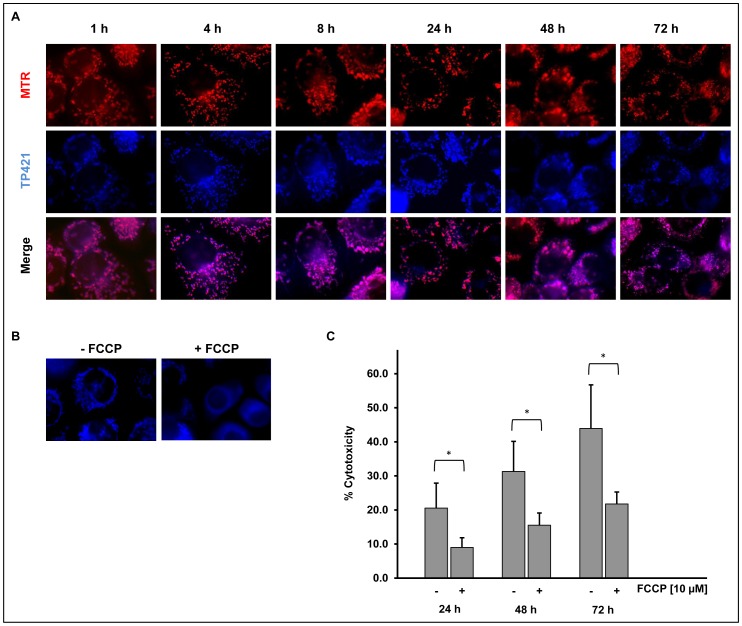
TP421 accumulates in mitochondria. (A) PANC-1 cells treated with TP421 and stained with MitoTracker Red (MTR) reveal extensive co-localization of TP421 and mitochondrial marker dye. (B) PANC-1 cells pretreated with FCCP show non-mitochondrial TP421 localization. (C) Reduced cytotoxicity of TP421 in MIA PaCa-2 cells pretreated with FCCP is seen at 24, 48 and 72 h. The data are mean ± SD from three independent experiments. * indicates p<0.01.

TP421 belongs to a class of compounds containing a TPP cation known to accumulate in mitochondria [27]. In order to confirm mitochondrial localization, PANC-1 cells were treated with 2 µM TP421 for various periods of time. Immediately before imaging, cells were co-stained with 200 nM MitoTracker Red dye to label the mitochondria. We observed significant co-localization persisting for up to 72 h (**Figure 5A**) indicating that TP421 indeed accumulated in mitochondria and was retained for a prolonged period of time. As secondary verification of mitochondrial localization we sought to determine the role of the mitochondrial transmembrane potential in the accumulation of TP421 in cells. Using the ionophore carbonyl cyanide-4-(trifluoromethoxy)phenylhydrazone (FCCP), commonly used to dissipate the mitochondrial transmembrane potential, we examined the pattern of TP421 fluorescence. We pretreated PANC-1 cells for 30 min with 10 µM FCCP followed by a 15 min incubation with TP421. Control cells received no FCCP and were exposed to 15 min TP421 only. In the presence of FCCP, we observed a distinctly diffuse TP421 fluorescence (**Figure 5B**) which did not resemble that of mitochondrial localization confirming that under normal conditions TP421 accumulated within mitochondria driven by the transmembrane potential. Since we had observed that 7-diethylamino-4-methylcoumarin was not cytotoxic to cells, we further explored the relationship between mitochondrial localization and TP421 cytotoxicity. We pretreated MIA PaCa-2 cells for 30 min with 10 µM FCCP then added TP421 for an additional 30 min. At the end of the treatment duration, we washed the cells and replaced the medium to remove the excess drug and incubated the cells for 24, 48 and 72 h and measured cell viability. In the presence of FCCP the cells were significantly protected from TP421 cytotoxicity (**Figure 5C**) indicating that localization to mitochondria was directly responsible for the action of our drug.

### TP421 Treatment Induces Mitochondrial and Cytosolic Accumulation of Reactive Oxygen Species

**Figure 6:**
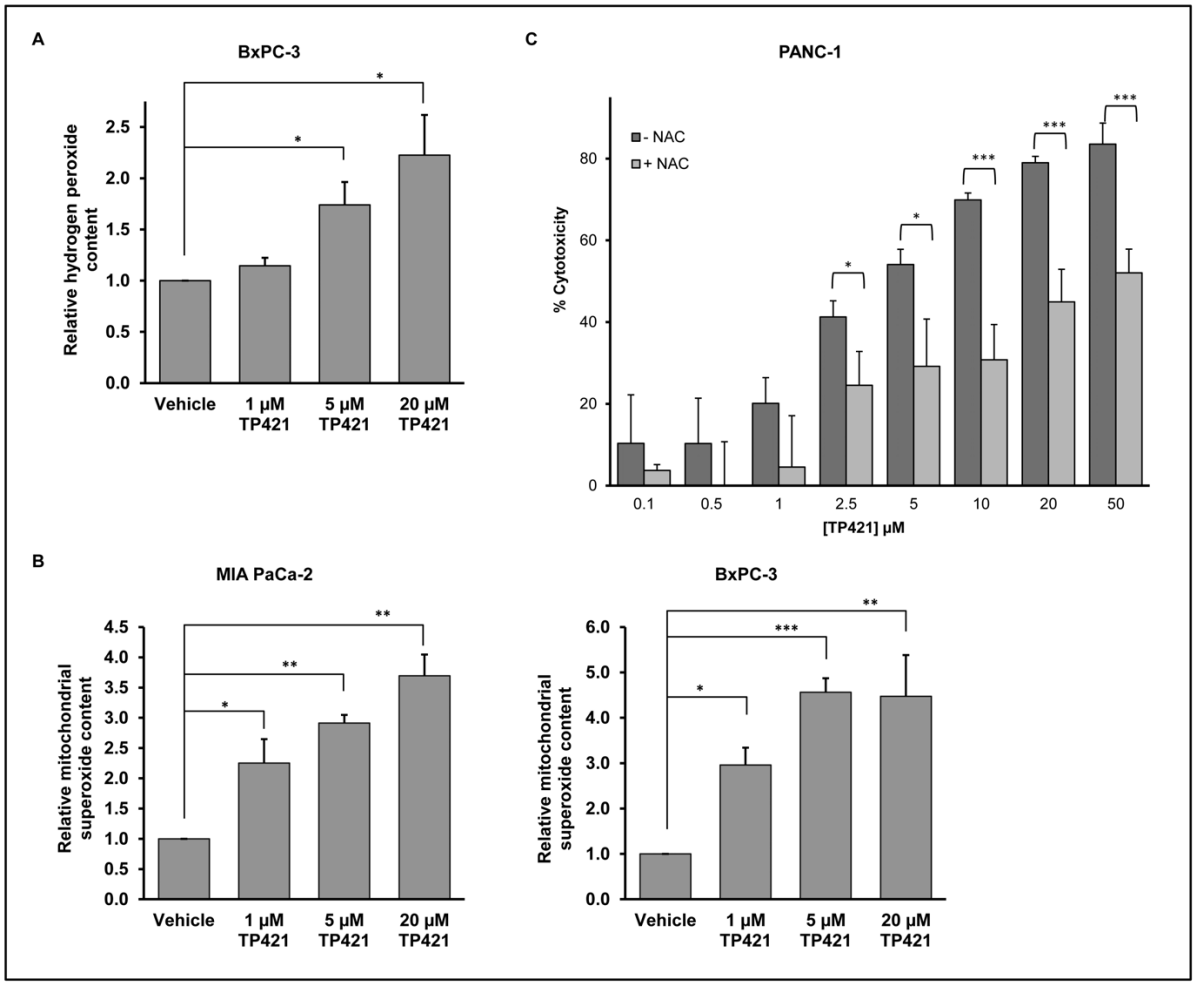
TP421 causes oxidative stress in pancreatic cancer cells. The effect of TP421 treatment on the levels of (A) H_2_O_2_ and (B) mitochondrial O_2_^−^ in BxPC-3 and MIA PaCa-2 cells were measured using the fluorescent probes Amplex Red and MitoSOX Red, respectively. (C) Effect of antioxidant pretreatment on the cytotoxicity of TP421 in PANC-1 cells. *, ** and *** indicate p<0.05, p<0.005 and p<0.001 respectively.

TP421 decreases mitochondrial respiratory capacity and increases superoxide (O_2_^−^) accumulation [26]. However, as compared to O_2_^−^, hydrogen peroxide (H_2_O_2_) is a relatively more stable and membrane-permeable ROS that can cause lipid, protein and DNA damage and participates in signal transduction [29]. Therefore, we examined the level of H_2_O_2_ in pancreatic cancer cells following TP421 treatment. MIA PaCa-2 and BxPC-3 cells were treated with increasing doses of TP421 for 4 hours followed by a brief incubation with 50 µM Amplex Red in the presence of 0.1 units/mL HRP. In the presence of HRP, Amplex Red reagent reacts with H_2_O_2_ in a 1∶1 stoichiometry to produce highly fluorescent resorufin. The resulting fluorescence intensity was measured at excitation and emission wavelengths of 540 nm and 590 nm, respectively. As expected, treatment resulted in a 2.5-fold increase in H_2_O_2_ levels in BxPC-3 at the highest concentration of TP421 with a good correlation between H_2_O_2_ levels and dose of TP421 used (**Figure 6A**). On the other hand, a similar increase in fluorescence could not be detected in MIA PaCa-2 cells (data not shown). To determine if the lack of H_2_O_2_ accumulation in these cells was due to an absence of O_2_^−^ generation, we used a MitoSOX red assay [26] to measure the level of mitochondrial O_2_^−^ in MIA PaCa-2 and compared it to BxPC-3. Both cells lines treated with increasing doses of TP421 for 4 h exhibited 3–5 fold increase in O_2_^−^ level (**Figure 6B**) and those levels remained high at 24 h (data not shown). This suggests that the lack of H_2_O_2_ accumulation observed in MIA PaCa-2 cells was not due to deficient O_2_^−^production in these cells.

As excessive ROS is known to lead to cell death, we further examined the potential protective role of an antioxidant. PANC-1 cells were pretreated with 15 mM *N*-acetyl-L- cysteine (NAC) for 2 h prior to addition of TP421. Following a 24 h incubation the extent of cytotoxicity was determined by MTT. Antioxidant pretreatment could protect cells from TP421 induced cytotoxicity by up to 35% (**Figure 6C**) and similar results were observed in MIA PaCa-2 and BxPC-3 cells (data not shown).

### TP421 Treatment Activates Stress-induced Pathways

**Figure 7:**
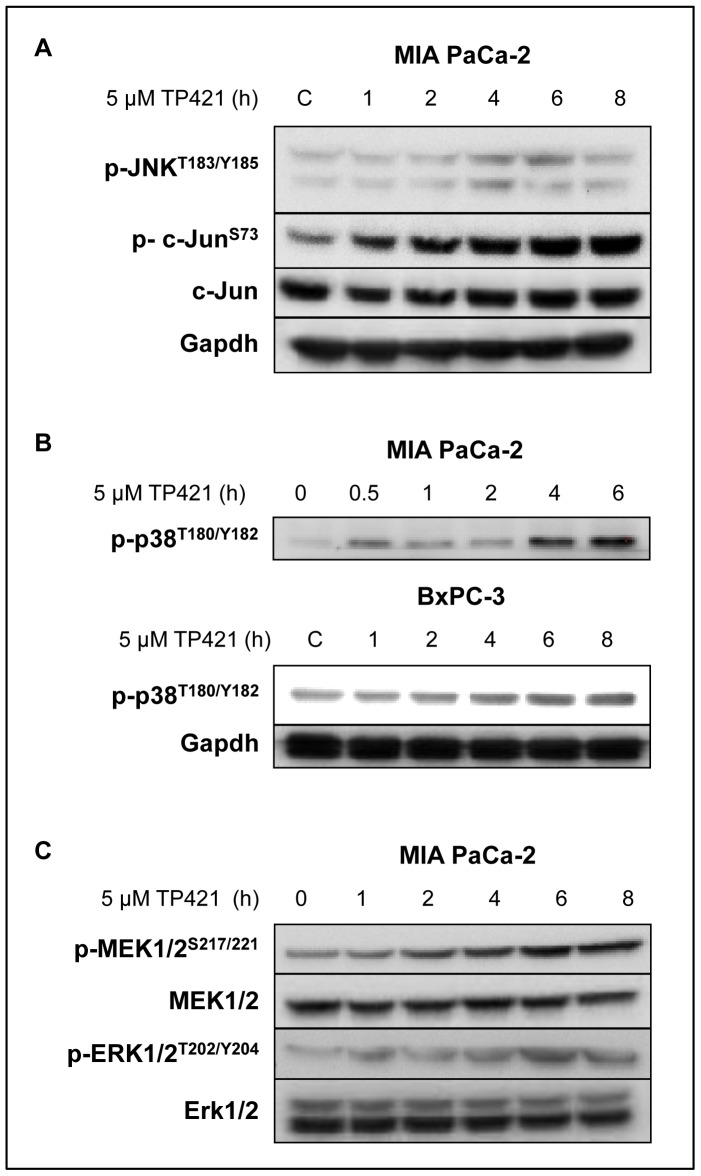
TP421 causes activation of stress induced signaling pathways and a sustained activation of MEK and ERK. Western blot analysis of the effect of TP421 treatment on the phosphorylation status of stress pathways. (A) MIA PaCa-2 cells were treated with 5 µM TP421 at increasing time points were probed for the phosphorylation of JNK1/2 and c-Jun. (B) MIA PaCa-2 and BxPC-3 cells were treated with 5 µM TP421 and probed for p-38 phosphorylation. Even loading for MIA PaCa-2 was verified using amido black total protein staining of membrane following transfer (data not shown) (C) MIA PaCa-2 cells were treated with 5 µM TP421 and probed for phosphorylation of MEK1/2 and Erk1/2.

Given the strong accumulation of ROS following TP421 treatment, we hypothesized that, cellular stress pathways JNK, p-38 and ERK, will be activated [30], [31], [32]. MIA PaCa-2 and BxPC-3 cells were treated with escalating doses of TP421 for 4 h or with 5 µM for 1–8 h and kinase activation was assessed by Western blotting. The TP421 treated MIA PaCa-2 cells had significant and sustained phosphorylation of c-Jun at S73 but only a transient increase in the phosphorylation of the upstream kinase, JNK1/2 (**Figure 7A**). Similarly, MIA PaCa-2 and BxPC-3 cells treated with TP421 exhibited enhanced phosphorylation of the stress induced p-38 kinase that increased over time (**Figure 7B**). Finally, we examined the activation state of the Erk1/2 kinase and its upstream kinase MEK1/2. TP421 induced a sustained increase in the activating phosphorylation of both kinases that appeared as early as 1–2 h and persisted for 8 h (**Figure 7C**).

### TP421 Induces Delayed DNA Damage and Apoptosis

**Figure 8:**
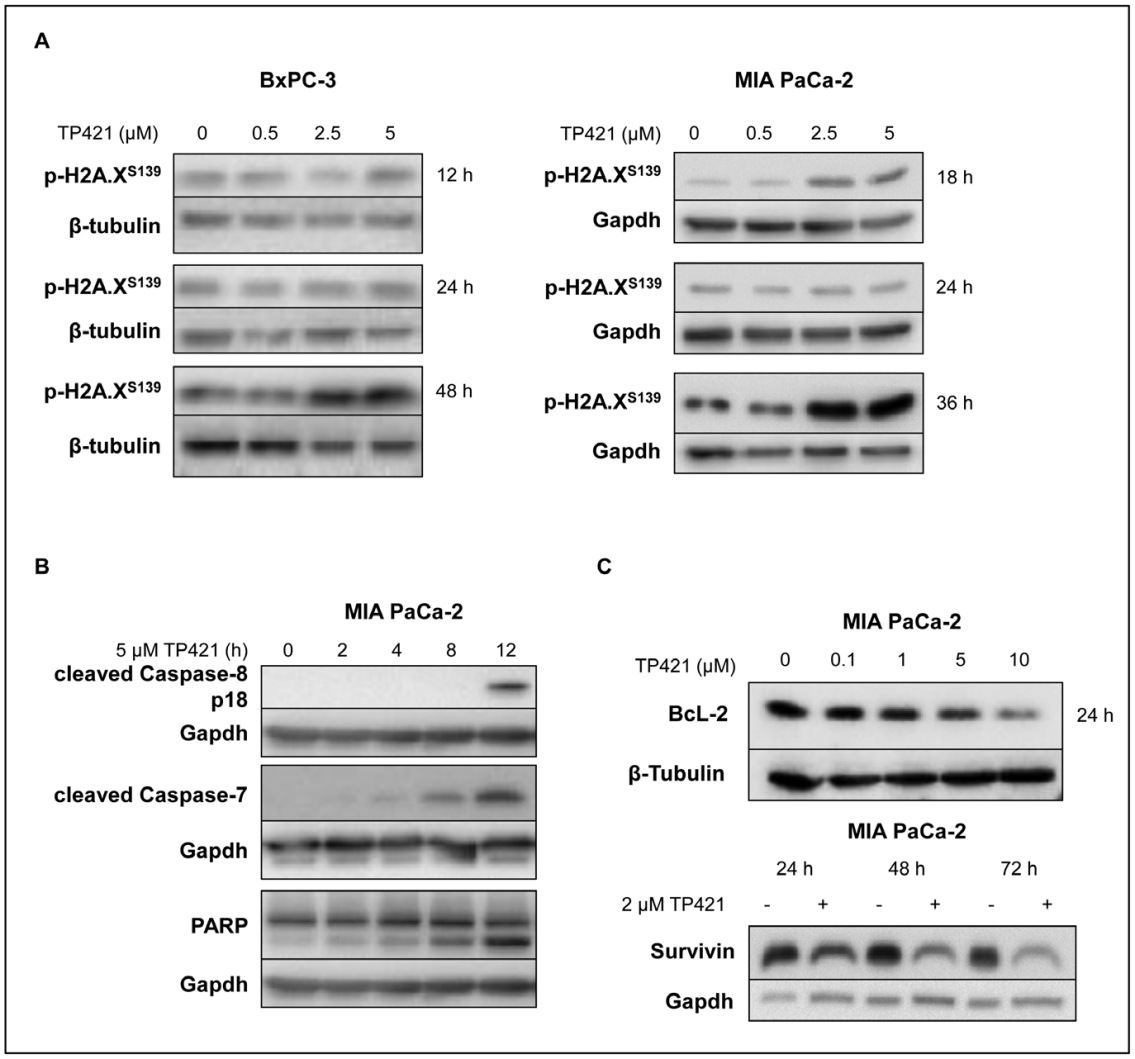
Effect of TP421 treatment on DNA damage and apoptosis induction. (A) BxPC-3 and MIA PaCa-2 cells treated with increasing concentrations of TP421 for 12–48 h were probed for the DNA damage marker phospho-H2A.X (S139). (B) MIA PaCa-2 cells treated with TP421 were probed for caspase activation and PARP-1 cleavage. (C) The effect of TP421 treatment on Bcl-2 and survivin levels was analyzed in MIA PaCa-2 cells.

Elevated cellular ROS can cause nuclear DNA damage leading to apoptosis and cell death. Having shown that TP421 can cause the accumulation of various forms of ROS originating from mitochondrial dysfunction, we examined whether this correlated with DNA damage in the nucleus. BxPC-3 and MIA PaCa-2 cells treated with TP421 were analyzed for phosphorylated H2A.X, a marker of DNA damage and double strand breaks [33]. There was a slight increase at 12–18 h, but a significant increase in the p-H2A.X did not occur until 36–48 h post treatment with at least 2.5 µM TP421 (**Figure 8A**). TP421 induced apoptosis as demonstrated by the cleavage of caspases-8 and 7, and PARP-1 as early as 8 h post treatment (**Figure 8B**). Extensive resistance to chemotherapy-induced apoptosis is a complicating factor in treating pancreatic cancer and has been linked to cellular overexpression of Bcl-2 and other apoptosis inhibitors [34]. Therefore we also examined the effect of TP421 treatment on the levels of Bcl-2 and survivin, a potent inhibitor of apoptosis. TP421 treatment at the indicated times could cause significant reduction of the total cellular level of both proteins (**Figure 8C**).

### TP421 Induces Accumulation of LC3B-II and p62, and Inhibition of Autophagy

**Figure 9:**
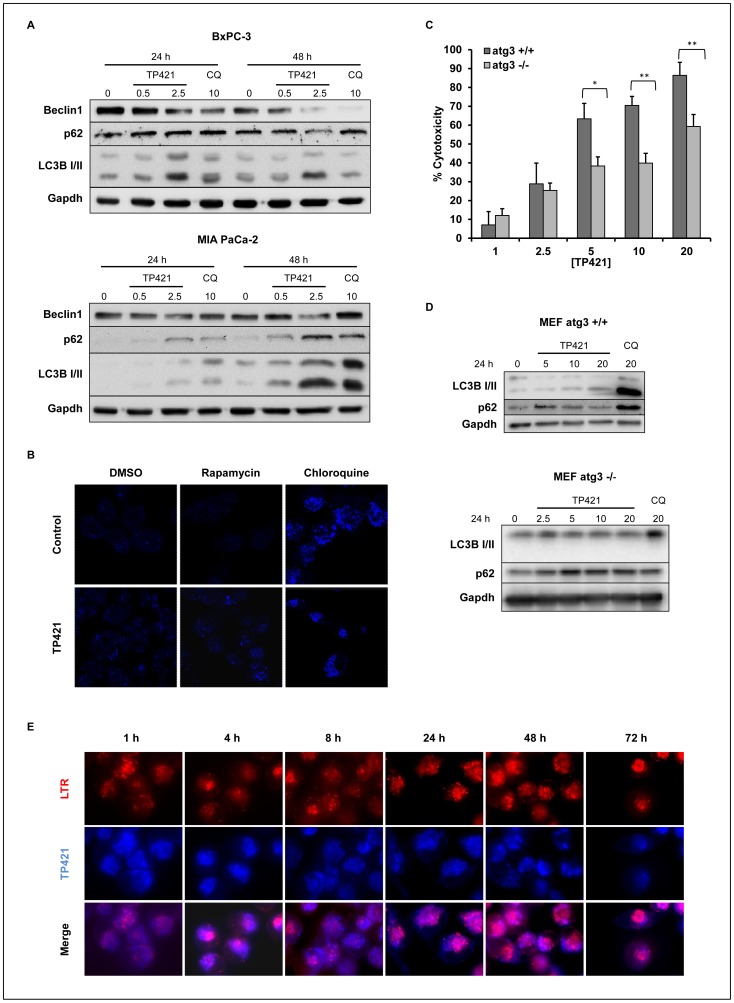
TP421 affects the autophagic response in pancreatic cancer cells. (A) BxPC-3 and MIA PaCa-2 cells were treated with 0.5 and 2.5 µM TP421 or chloroquine for 24 and 48 h and probed for Beclin1, p62, and LC3B-I/II. (B) LC3B puncta formation in MIA PaCa-2 cells treated with TP421 for 18 h alone or in combination with rapamycin or chloroquine. (C) MEF cells competent in autophagy (atg3+/+) and deficient in autophagy (atg3−/−) were treated with increasing concentrations of TP421 and percent cytotoxicity was determined by MTT. The data are mean ± SD from three independent experiments. * and ** indicate p<0.05 and p<0.001 respectively. (D) MEF atg3+/+ and −/− cells treated with TP421 for 24 h and probed for autophagy proteins LC3B-I/II and p62. (E) MIA PaCa-2 cells treated with TP421 for increasing durations of time and co-stained with LysoTracker Red (LTR).

Having established the direct effect of TP421 on mitochondria and because of the reliance of pancreatic cancer on autophagy to maintain a healthy mitochondrial pool, we investigated the effects of TP421 on autophagy. Whole cell lysates from MIA PaCa-2 and BxPC-3 cells treated with TP421 for 24 and 48 h were analyzed for the markers of autophagy LC3B-I/II, p62, and Beclin-1. As a control, cells were treated with the late stage autophagy inhibitor chloroquine that inhibits fusion of phagosomes and lysosomes thereby accumulating lipidated LC3B-II in cells. TP421 treatment in both cell lines caused a dose and time dependent increase in the lower migrating band of LC3B, the lipidated form that associates with autophagosome membranes (**Figure 9A**). This can indicate an increase in autophagic flux or inhibition of autophagy and therefore accumulation of autophagosome bound LC3B-II. In order to differentiate between these two scenarios, we examined the levels of p62, a ubiquitin binding protein that is selectively degraded during autophagy and its levels increase only if autophagy is inhibited [35]. Interestingly, TP421 treatment caused a robust accumulation of p62 in MIA PaCa-2 cells, suggesting that the increase in lipidated LC3B was indicative of inhibition of autophagy. Accordingly, the levels of Beclin-1, a protein upstream of both LC3B and p62 in the autophagy pathway that contributes to the initiation of autophagosome formation, decreased with TP421 treatment in a time and dose-dependent manner and therefore correlated with a decrease in autophagy. On the other hand, p62 levels in BxPC-3 cells decreased by 48 h following TP421 treatment indicating a possible induction of autophagy in these cells, albeit only moderately.

To further confirm inhibition of autophagy in MIA PaCa-2 cells, we fixed and stained cells treated with TP421 alone or in combination with an autophagy inducer (i.e. rapamycin) or autophagy inhibitor (i.e. chloroquine) and observed the level of puncta staining with LC3B antibody **(** **Figure 9B** **)**. TP421 alone could induce accumulation of LC3B puncta and this was further increased in the presence of rapamycin confirming TP421’s inhibition of autophagy in these cells. Cells treated with TP421 and chloroquine resembled chloroquine only treated cells.

The fact that in MIA PaCa-2 cells, autophagy was inhibited rather than stimulated by TP421 prompted us to examine whether autophagy proficient cells would show greater sensitivity to our compound as compared to autophagy deficient ones. When atg3 null and wild-type MEF cells were treated with increasing concentrations of TP421 for 24 h, we found that the autophagy deficient cell line was more resistant to TP421 treatment as compared to the wildtype (**Figure 9C**). Subsequently, the effect of TP421 on the levels of LC3B-I/II and p62 in the MEF atg3+/+ cell line was assessed to determine if autophagy was being inhibited or induced in these cells. TP421 caused inhibition of autophagy at higher concentration (20 µM), as can be seen by an increase in LC3B-II accompanied with maintenance of basal p62 levels (**Figure 9D**). Interestingly, at the lower concentration (5 µM) there was some accumulation of p62, but with undetectable change in the level of lipidated LC3B, which led us to consider that the increase in p62 protein might be occurring by another means independently from autophagy inhibition. To explore that further, we examined the effect of TP421 treatment on p62 levels in the autophagy deficient cell line but found that p62 levels were unchanged (**Figure 9D**).

To assess if TP421’s effect on autophagy could be modulated in part by a fraction of its intracellular pool localizing at or interacting with lysosomes, we imaged MIA PaCa-2 cells treated with 2 µM TP421 for increasing durations of time and observed its location relative to the lysosome specific LysoTracker red dye. Interestingly, TP421 was not found to localize to lysosomal compartments even as late as 72 h post treatment (**Figure 9E**).

### TP421 Inhibits Src-FAK Mediated Cell Migration

**Figure 10:**
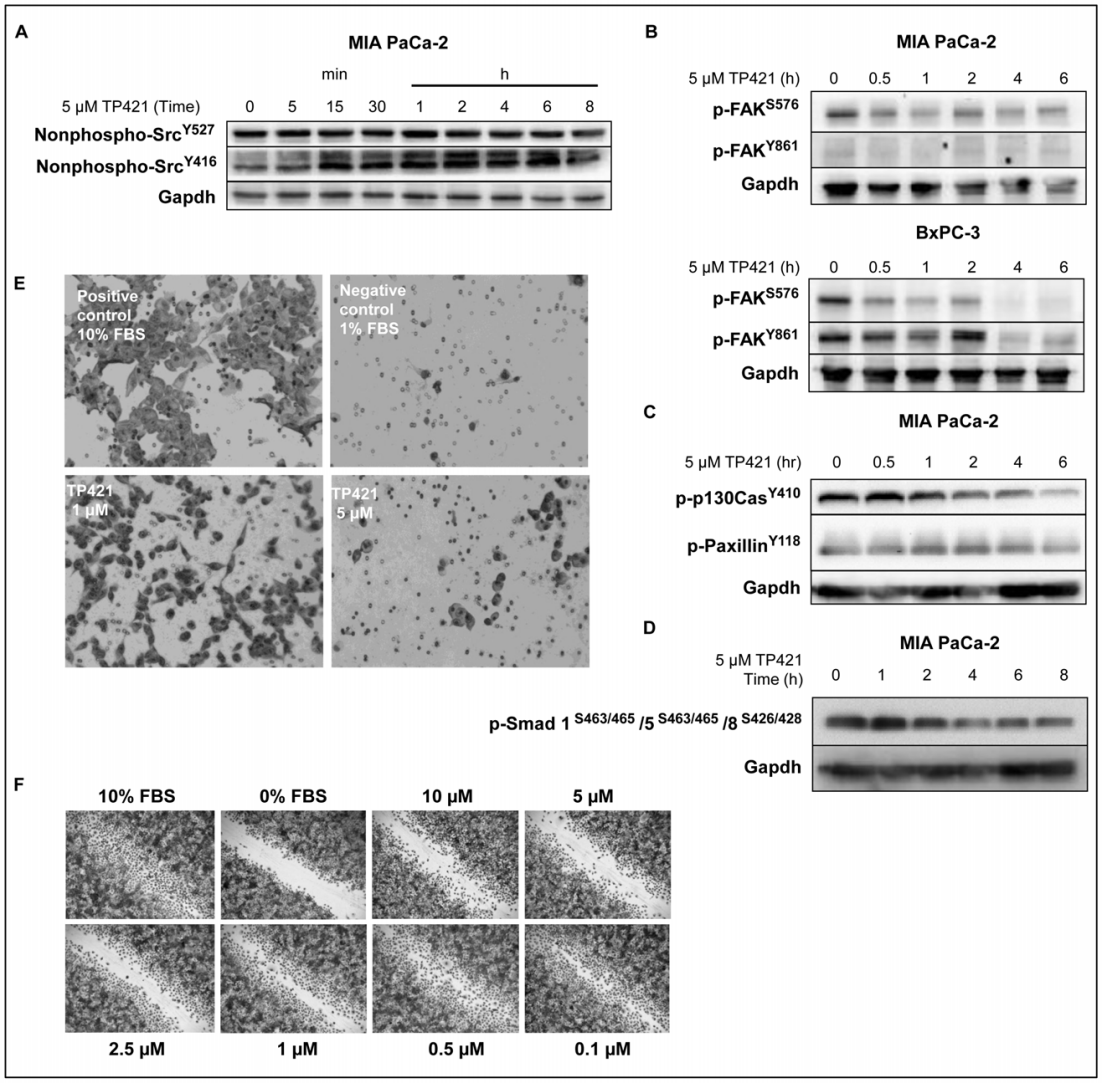
TP421 decreases signaling via Src-FAK and inhibits cell migration. (A) MIA PaCa-2 cells treated with 5 µM TP421 for indicated time and probed for de-activating phosphorylation (Y527) and the activating phosphorylation (Y416) of Src. (B) MIA PaCa-2 and BxPC-3 cells were treated with 5 µM TP421 and probed for phosphorylation of FAK. (C) Effect of 5 µM TP421 treatment on phosphorylation status of p130Cas and Paxillin proteins downstream of Src activation. (D) 5 µM TP421 treatment decreases the phosphorylation of Smad1/5/8 in MIA PaCa-2 cells. (E) Effect of 24 h TP421 treatment of serum starved MIA PaCa-2 cells on their ability to migrate through a Boyden Chamber setup. (F) TP421 treated PANC-1 cells are inhibited from migrating into denuded area of the wound.

One of the reasons pancreatic cancer is very difficult to treat is that the tumor is highly invasive and metastatic. The Src kinase – focal adhesion kinase (FAK) pathway plays an important role in mediating cell motility but is also involved in regulating cell survival, proliferation and differentiation [36]. Furthermore, Src levels have been shown to be elevated in many cancers including pancreatic cancer [37]. This observation prompted investigations of Src and FAK inhibitors that showed anticancer activity as well as ability to inhibit migration, invasion and anchorage-independent survival of pancreatic cancer cells [37], [38], [39]. Accordingly, we sought to determine the effect of TP421 on key phosphorylation events in the Src-FAK signaling. Western blot analysis of the phosphorylation of Src kinase revealed a decrease in the phosphorylation status of the Y416 residue that is the activating phosphorylation for Src activity (**Figure 10A**). There was a corresponding decrease in phosphorylation of FAK at residues S576 and Y861 that are downstream of activated Src and are directly phosphorylated by it (**Figure 10B**). We also observed a decrease in phosphorylation of p130Cas and paxillin (**Figure** **10C**) that are members of a complex of focal adhesion-associated proteins and are phosphorylated in response to activated Src-FAK [36]. Additionally, our findings indicated that TP421 could induce de-phosphorylation of Smads 1, 5 and 8 (**Figure 10D**) that have been shown to be frequently hyper-phosphorylated in precursor lesions of PDAC [40] and to contribute to cancer metastasis [41].

Because we found a robust decrease in the phosphorylation of Src, FAK and downstream targets, we proceeded to determine if TP421 could inhibit the migration of MIA PaCa-2 cells in a Boyden Chamber assay when stimulated with FBS. Migration of serum starved cells treated with 5 µM TP421 was significantly lower as compared to the stimulated un-treated control (**Figure 10E**). This difference was not due to cytotoxicity as cells treated in parallel and analyzed via MTT showed approximately 70% viability at a 5 µM TP421 dose (data not shown). Wound closure in PANC-1 treated cells was equally inhibited by TP421 (**Figure 10F**).

## Discussion

Herein we report on the anticancer mechanisms of our novel small molecule TP421, which by virtue of its TPP moiety, was specifically accumulated in mitochondria. Importantly, TP421 exhibits sub-micromolar efficacy in all pancreatic cancer cell lines we tested regardless of their subtype classification indicating a potential clinical utility in diverse patient populations. Furthermore, TP421 efficacy was independent of k-ras mutation as it was equally cytotoxic in the k-ras mutant cell lines and the wild type cell line, BxPC-3. More importantly, TP421 was highly selective towards cancer cells over normal fibroblast cells. Notably, the mitochondrial targeting moeity was essential to the efficacy of TP421 as the structurally identical 7-diethylamino-4-methylcoumarin lacking the TPP moiety was inactive. Additionally, when accumulation of TP421 in mitochondria was hindered by the use of the uncoupler FCCP to depolarize the mitochondria, the cells were protected from TP421 cytotoxicity. It has been previously demonstrated that specific targeting of apoptotic compounds to the mitochondria, via TPP conjugation, can greatly enhance potency which is in line with our findings [22], [20]. Lipophilic cations, including TPP, do not impart cytotoxicity as evidenced by their development as tumor targeted PET tracers and targeting moieties for mitochondria protective antioxidants [42], [43]. In light of this, our results validate that localization to the mitochondria is responsible for the cytotoxic action of TP421 and suggest that its preferential accumulation within tumor mitochondria will aid its clinical usefulness as a safe and effective anti-neoplastic agent. Furthermore, the ability of TP421 to induce cell growth inhibition following very short treatment durations demonstrates its capacity to rapidly accumulate within mitochondria thereby affecting cell survival over prolonged time and supports its clinical efficacy where it can be dosed intermittently and at lower concentrations.

To better understand the mechanism by which TP421 acts to cause cytotoxicity, we extended our previous findings regarding mitochondrial respiratory dysfunction induced by TP421 by examining the levels of ROS in the mitochondria and cytosol. O_2_^−^ is mostly generated at complexes I and III of the OXPHOS chain as a consequence of electron leakage and inhibitors of these complexes can also cause large amounts of O_2_^−^ to be produced [44]. Therefore increase in mitochondrial O_2_^−^ levels can be indicative of inhibition of one of these sites along the OXPHOS chain however other enzyme reactions can also release O_2_^−^ in mitochondria but are poorly characterized. While not examined in this study, we speculate that TP421 may target one of these complexes supported by the findings that TP421 inhibits mitochondrial oxygen consumption and induces prolonged O_2_^−^ accumulation. TP421 produced a sustained increase of mitochondrial O_2_^−^ in both MIA PaCa-2 and BxPC-3 cell lines but, only the latter showed a concomitant increase in H_2_O_2_ levels. Under normal cellular homeostatic conditions O_2_^−^ is rapidly converted to H_2_O_2_ by the action of superoxide dismutases (SOD) that are present in the mitochondria (MnSOD), cytosol (CuZnSOD) and extracellularly (EcSOD). However, cancer cells frequently have aberrant antioxidant mechanisms including diminished SOD expression which might account for the discrepancy in H_2_O_2_ accumulation we observed [45], [46]. However, as both BxPC-3 and MIA PaCa-2 cells are reported to have similar levels of SOD enzyme activities it does not explain the apparent impaired conversion of O_2_^−^ to H_2_O_2_ in the latter cell line [47]. Another possibility could be that MIA PaCa-2 exhibit a stronger inducible antioxidant response as compared to BxPC-3 leading to more efficient H_2_O_2_ detoxification. During oxidative stress the transcription factor NF-E2-related factor 2 (Nrf2) translocates to the nucleus and binds to antioxidant response elements (ARE) to activate transcription of target genes involved in reducing intracellular ROS [48]. Several pancreatic cancer cell lines including MIA PaCa-2 have documented Nrf2 overexpression [49] and intriguingly Nrf2 maybe up-regulated in these cells by mutant K-ras which has been demonstrated to increase Nrf2 transcription and lead to an elevated antioxidant program [50]. Given that BxPC-3 is a wildtype K-ras cell line while MIA PaCa-2 harbors a mutation in codon 12, a variable Nrf2 response might explain the difference in H_2_O_2_ levels we detected [51]. Nevertheless, the increase in mitochondrial ROS following TP421 treatment is expected to contribute to induction of apoptosis and programmed cell death. In fact, activation of caspase-7 and 8 followed by PARP-1 cleavage were observed and caspase-8 has been shown to be activated by O_2_^−^oxidative stress [52] and prolonged ERK1/2 activation [53] both of which are observed in our treated cells. It seems that TP421 can initiate apoptosis induction via its direct effects on mitochondria as caspase activation was an early response occurring even before DNA damage could be detected. Furthermore, apoptosis was accompanied by significant reduction in the protein levels of the apoptosis inhibitors Bcl-2 and survivin. This diminution in survivin is possibly related to the strong activation of p38 we observed. It has been previously reported that p38 activation can cause reduction in survivin levels and that this effect could be prevented by a specific p38 inhibitor [54], [55]. As survivin expression has been associated with poor prognostic outcome and resistance to chemotherapy there is interest in targeting its degradation as a novel treatment for pancreatic cancer [56]. Bcl-2 overexpression is also another common resistance mechanism precluding gemcitabine efficacy in pancreatic cancer [57]. Therefore, it is promising that TP421 induces decreased Bcl-2 and survivin levels and provides a rationale for future exploration of combination treatment with gemcitabine.

Following oxidative stress induction, TP421 treatment produced sustained activation of JNK, p38 and Erk1/2 pathways. Recent reports indicate that mitochondrial pools of each of these kinases exist and are sensitive to mitochondrially generated O_2_^−^ [58], [59], [60]. Interestingly, activation of JNK by elevated superoxide in cells may play a role in further amplifying the ROS produced at sites within mitochondria [61]. It has also been suggested that mitochondrial activation of Erk1/2 in response to oxidative stress can suppress mitochondrial respiration and ATP production [62] thereby providing a link between the TP421 induced Erk1/2 activation and mitochondrial dysfunction we observe. Furthermore, prolonged robust activation of ERK1/2 is known to induce cell cycle arrest in the G1 phase [63]. Our results correlate well with this finding as TP421 could potently arrest multiple pancreatic cancer cell lines in the G_0_/G_1_ phase thereby hindering their proliferation.

In addition to induction of apoptosis, it was observed that TP421 could inhibit autophagy in pancreatic cancer cells as observed by LC3B-II and p62 accumulation. This is particularly appealing because of the important role autophagy has been shown to play in supporting pancreatic cancer survival and proliferation and suggests that TP421 could be especially effective for treating these tumors in patients. Interestingly, caspase 7 has recently been shown to affect autophagy regulation via its action on TDP-43, a protein responsible for the maintenance of atg7 mRNA levels [64]. Apparently, following caspase 7 mediated cleavage of TDP-43, atg7 protein levels decrease and LC3B-II is accumulated, indicating inhibition of autophagy [64], [65]. Considering that TP421 causes casapse-7 activation, it is an intriguing possibility that TP421 might achieve inhibition of autophagy via caspase-7 mediated degradation of TDP-43.

It is worthwhile to note that p62 levels can also be increased in cells undergoing oxidative stress independently of autophagy modulation. ROS disruption of a KEAP1-Nrf2 complex stabilizes Nrf2 levels allowing it to initiate its transcriptional oxidative stress response program which includes induction of the p62 gene [66]. Alternatively, accumulated p62 levels due to autophagy interruption can disrupt KEAP1-Nrf2 complex via p62 competitive binding to KEAP1 [67]. The resulting Nrf2 activation would further increase p62 levels [67]. Given that TP421 treatment failed to induce significant accumulation of p62 in autophagy deficient MEF cells (ATG3−/−) we are confident that accumulation of p62 is indeed the result of autophagy inhibition.

Importantly, TP421 could inhibit the migration of MIA PaCa-2 cells *in vitro*, which may be mediated by its ability to cause de-phosphorylation of multiple proteins involved in adhesion and motility signaling including FAK, Src and p130Cas. Frequently in PDAC, FAK is activated and its levels are negatively correlated with survival [68]. FAK phosphorylation has also been identified as a cause for chemoresistance to gemcitabine [69], supporting its inhibition as a means to overcoming resistance to cell death.

The manifold consequences of TP421 treatment including inhibition of mitochondrial respiration, autophagy and cell motility highlight the utility of this class of compounds at affecting essential cancer cell processes. The prospective clinical usefulness of our novel class of mitochondrial-targeted agents for treating pancreatic cancer merits their further development.

## Supporting Information

Figure S1 **Structures of TP compounds and the related non-TPP tagged 7-Diethylamino-4-methylcoumarin compound used in this study.**
(TIF)
