# Supplementary figures and images for: Mechanistic Evaluation of a Novel Small Molecule Targeting Mitochondria in Pancreatic Cancer Cells
Source: PLoS One. 2013 Jan 21;8(1):e54346. doi: 10.1371/journal.pone.0054346 (PMC3549929; doi:10.1371/journal.pone.0054346)

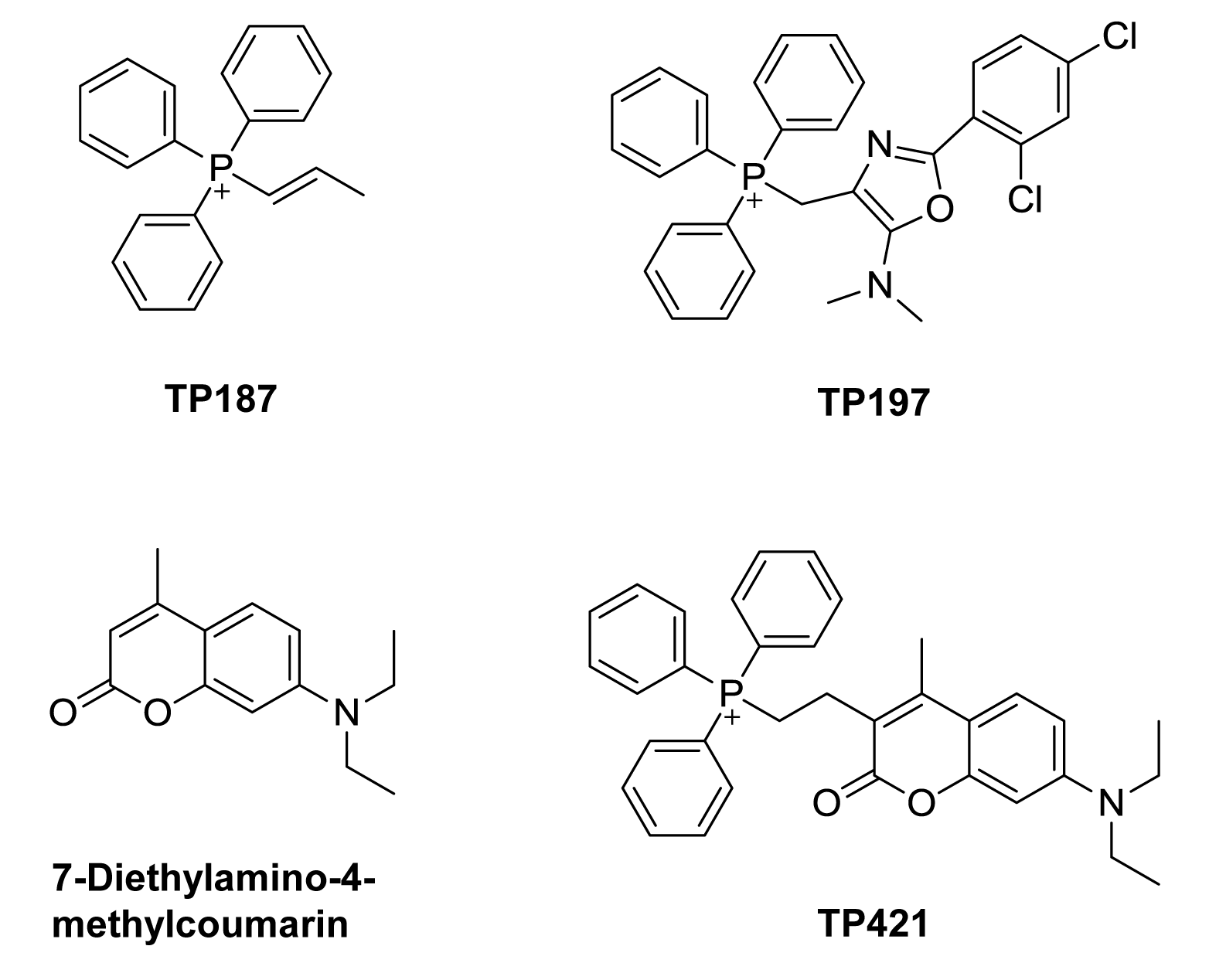

Supplement: Figure S1 — Structures of TP compounds and the related non-TPP tagged 7-Diethylamino-4-methylcoumarin compound used in this study. (TIF) [file pone.0054346.s001.tif]
